# Supplementary material for: Physical and mental health in adolescence: novel insights from a transdiagnostic examination of FitBit data in the ABCD study
Source: Transl Psychiatry. 2024 Feb 3;14:75. doi: 10.1038/s41398-024-02794-2 (PMC10837202; doi:10.1038/s41398-024-02794-2)
Supplement: Supplementary file 1 — Supplemental Information [file 41398_2024_2794_MOESM1_ESM.docx]

**Supplemental Material**

**Demographic Comparison of Current Study Subsample.** Representativeness of the sample was assessed by comparing the current analytic sample to (1) the whole ABCD sample, (2) the United States demographics from the American Community Survey (ACS), and (3) the subsample of individuals that were excluded for not meeting the minimum wear time.^40,51^ There were no significant differences in the distribution of sex assigned at birth or household income compared to the ABCD, the ACS sample, or the excluded fitbit sample (*χ*^2^’s<2.05, *p*>.15, *OR*’s=0.97-1.04). The current study sample underrepresented the lowest income category compared to the ABCD (*OR*=0.74; *χ*^2^(2)=110.52, *p*<2.2e^-16^) and the excluded fitbit sample (*OR*=0.74; *χ*^2^(2)= 60.448, *p*=7.48e^-14^). The current study sample underrepresented in the lowest income category (*OR*=0.54) and overrepresented the highest income category (OR=1.68) compared to the ACS sample (*χ*^2^(2)=1097.2, *p*<2.2e^-16^), The current sample overrepresented white (OR’s=1.11-1.38) and multi-racial (OR’s=1.24-2.71) individuals and underrepresented black (OR’s=0.58-0.71) compared to the ABCD (*χ*^2^(3)= 215.34, *p*<2.2e^-16^), the ACS sample (*χ*^2^(3)= 669.06, *p*<2.2e-16), and the excluded fitbit sample (*χ*^2^(3)= 97.739, *p*=2.2e-16).

**Comparison of Excluded Individuals to the ABCD Study.** The sample excluded from the fitbit analyses on the basis of the time spent wearing the device did not differ in demographic distribution from the whole ABCD sample in terms of sex (*χ*^2^(1)=1.0712, *p*=.3007), race(*χ*^2^(2)=0.54524, *p*=.7614), or household income (*χ*^2^(3)= 0.89351, *p*=0.8826).

Supplemental Table 1. Demographic Samples Comparison

| Demographic Parameter | Current Subsample | ABCD Study | American Community Survey | ABCD FitBit Excluded |
| --- | --- | --- | --- | --- |
| Sex at Birth | % |  |  |  |
| Female | 48.41% | 47.80% | 48.80% | 46.68% |
| Male | 51.59% | 52.20% | 51.20% | 53.32% |
| Race | % |  |  |  |
| Asian/American Indian/Alaska Native/Native Hawaiian/Pacific Islander | 3.07% | 3.20% | 5.90% | 1.78% |
| Black | 9.47% | 15.10% | 13.40% | 16.41% |
| Multiple | 11.38% | 9.20% | 4.20% | 12.40% |
| White | 72.21% | 52.20% | 52.40% | 65.10% |
|  |  |  |  |  |
| Household Income | % |  |  |  |
| <50K | 23.18% | 31.20% | 43.20% | 31.19% |
| >=50K & <100K | 30.98% | 28.10% | 29.50% | 28.75% |
| >=100K | 45.83% | 40.70% | 27.30% | 40.06% |

**Modeling Containing Correction for the average daily wear time.** Fitness metrics were predicted by current symptoms in a multilevel model that accounted for the random effects of individuals and relatedness and the fixed effects of age, sex, average daily time recording data, socioeconomic status, and body mass index.

Supplemental Table 2. Modeling Containing Correction for the average daily wear time

| Fitness Metric | Parameter | t-value | p-value | Bonferroni |
| --- | --- | --- | --- | --- |
| Sedentary Activity | PLE Severity | 5.087 | 3.78E-07 | * |
|  | Internalizing Symptoms | -0.054 | 9.57E-01 |  |
|  | Externalizing Symptoms | -0.081 | 0.935167 |  |
|  | Age at Interview | 6.898 | 6.06E-12 | * |
|  | Average Daily Data (min) | 17.596 | < 2e-16 | * |
|  | Sex at Birth | 0.799 | 4.24E-01 |  |
|  | BMI | 5.628 | 1.94E-08 | * |
|  | Income (>=100k vs >50k) | -4.381 | 1.21E-05 | * |
|  | Income (50k-100k vs >50k) | -3.641 | 2.74E-04 | * |
|  |  |  |  |  |
| Moderate Activity | PLE Severity | -2.53 | 1.14E-02 | * |
|  | Internalizing Symptoms | -6.34 | 2.58E-10 | * |
|  | Externalizing Symptoms | 2.2 | 0.02811 |  |
|  | Age at Baseline | 0.608 | 0.54315 |  |
|  | Average Daily Data (min) | -5.097 | 3.58E-07 |  |
|  | Sex at Birth | 33.113 | < 2e-16 | * |
|  | BMI | 18.821 | < 2e-16 | * |
|  | Income (>=100k vs >50k) | 6.443 | 1.31E-10 | * |
|  | Income (50k-100k vs >50k) | 2.716 | 0.00664 | * |
|  |  |  |  |  |
| Fitness Metric | Parameter | t-value | p-value | Bonferroni |
| Resting Heart Rate (RHR) | PLE Severity | 1.905 | 5.68E-02 |  |
|  | Depression Symptoms | 3.055 | 2.26E-03 | * |
|  | Anxiety Symptoms | 1.468 | 0.14206 |  |
|  | Externalizing Symptoms | 1.324 | 0.18546 |  |
|  | Age at Interview | -9.67 | < 2e-16 | * |
|  | Average Daily Data (min) | 0.49 | 0.62439 |  |
|  | Sex at Birth | -12.425 | < 2e-16 | * |
|  | BMI | 12.66 | < 2e-16 | * |
|  | Income (>=100k vs >50k) | -5.76 | 8.83E-09 | * |
|  | Income (50k-100k vs >50k) | -2.26 | 0.02373 |  |
| Moderate Activity | PLE Severity | -2.345 | 1.91E-02 |  |
|  | Depression Symptoms | -3.89 | 1.02E-04 | * |
|  | Anxiety Symptoms | -2.588 | 0.009688 | * |
|  | Externalizing Symptoms | 2.142 | 0.03224 |  |
|  | Age at Interview | 0.745 | 0.45613 |  |
|  | Average Daily Data (min) | -5.058 | 4.40E-07 | * |
|  | Sex at Birth | 33.135 | < 2e-16 | * |
|  | BMI | 18.792 | < 2e-16 | * |
|  | Income (>=100k vs >50k) | 6.208 | 2.82E-11 | * |
|  | Income (50k-100k vs >50k) | 2.47 | 0.00852 | * |

Analytic Code

**Modeling Containing Proportion of daily wear time.** Fitness metrics were predicted by current symptoms in a multilevel model that accounted for the random effects of individuals and relatedness and the fixed effects of age, sex, socioeconomic status, and body mass index.

Supplemental Table 3. Proportion of recorded time spent sedentary and moderate to intense

| Fitness Metric | Parameter | t-value | p-value | Bonferroni |
| --- | --- | --- | --- | --- |
| Sedentary Activity | PLE Severity | 4.348 | 1.40E-05 | * |
|  | Internalizing Symptoms | -0.019 | 9.85E-01 |  |
|  | Externalizing Symptoms | -0.214 | 0.83036 |  |
|  | Age at Interview | 6.898 | 6.06E-12 | * |
|  | Sex at Birth | 0.715 | 4.75E-01 |  |
|  | BMI | 5.519 | 3.60E-08 | * |
|  | Income (>=100k vs >50k) | -3.205 | 1.21E-05 | * |
|  | Income (50k-100k vs >50k) | -3.641 | 2.74E-04 | * |
|  |  |  |  |  |
| Moderate Activity | PLE Severity | -2.789 | 1.14E-02 | * |
|  | Internalizing Symptoms | -6.23 | 2.58E-10 | * |
|  | Externalizing Symptoms | 2.03 | 0.02811 |  |
|  | Age at Baseline | 0.608 | 0.54315 |  |
|  | Sex at Birth | 32.31 | < 2e-16 | * |
|  | BMI | 17.98 | < 2e-16 | * |
|  | Income (>=100k vs >50k) | 7.45 | 1.12E-13 | * |
|  | Income (50k-100k vs >50k) | 3 | 0.00272 | * |
|  |  |  |  |  |
| Fitness Metric | Parameter | t-value | p-value | Bonferroni |
| Resting Heart Rate (RHR) | PLE Severity | 4.149 | 3.40E-05 |  |
|  | Depression Symptoms | 2.808 | 5.01E-03 | * |
|  | Anxiety Symptoms | -1.654 | 0.09825 | * |
|  | Externalizing Symptoms | -1.239 | 0.21558 |  |
|  | Age at Interview | -9.67 | <2e-16 |  |
|  | Sex at Birth | 0.708 | 0.47923 |  |
|  | BMI | 5.417 | 6.36E-08 |  |
|  | Income (>=100k vs >50k) | -3.19 | 1.43E-03 | * |
|  | Income (50k-100k vs >50k) | -3.023 | 0.00252 | * |
| Moderate Activity | PLE Severity | -2.589 | 9.66E-03 | * |
|  | Depression Symptoms | -4.23 | 2.38E-05 | * |
|  | Anxiety Symptoms | -2.274 | 0.02298 | * |
|  | Externalizing Symptoms | 2.095 | 0.03626 |  |
|  | Age at Interview | 0.745 | 0.45613 |  |
|  | Sex at Birth | 32.35 | <2e-16 |  |
|  | BMI | 17.98 | <2e-16 |  |
|  | Income (>=100k vs >50k) | 7.228 | 5.85E-13 |  |
|  | Income (50k-100k vs >50k) | 2.759 | 0.00582 | * |

**Main Findings Crosstab by Sex and Age.** This time period is a dynamic period of metabolic and sexually dimorphic developmental change

#### Fit Bit Metrics to predict symptom trajectories

library(readr)

library(dplyr)

library(lme4)

library(lmerTest)

library(psych)

library(ggplot2)

library(gridExtra)

library(car)

library(tidyr)

#

#### Data cleaning and compiling ####

## Fitbit weekly Averages Downloaded June 22 nd from NDA

#

#

# abcd_fbwpas01.txt

ABCD_weekly_activity_scales <- read_csv("abcd_fbwpas01.csv", na = "NA")

#removing folks who were excluded based on ABCD standards

ABCD_weekly_activity_scales%>%filter(ABCD_weekly_activity_scales$fit_ss_meet_abcd_rule=="1")->ABCD_weekly_activity_scales

#filtering for the estimates based on all available days

ABCD_weekly_activity_scales%>%filter(ABCD_weekly_activity_scales$fit_ss_day_weekend_ind=="2")->ABCD_weekly_activity_scales_alldays

#define factors and numeric variables

ABCD_weekly_activity_scales_alldays$subjectkey<-as.factor(ABCD_weekly_activity_scales_alldays$subjectkey)

ABCD_weekly_activity_scales_alldays$fit_ss_day_wkno<-as.factor(ABCD_weekly_activity_scales_alldays$fit_ss_day_wkno)

ABCD_weekly_activity_scales_alldays$sex<-as.factor(ABCD_weekly_activity_scales_alldays$sex)

ABCD_weekly_activity_scales_alldays$interview_age<-as.numeric(ABCD_weekly_activity_scales_alldays$interview_age)

ABCD_weekly_activity_scales_alldays$fit_ss_perday_sedentarymin<-as.numeric(ABCD_weekly_activity_scales_alldays$fit_ss_perday_sedentarymin)

ABCD_weekly_activity_scales_alldays$fit_ss_perday_veryactivemin<-as.numeric(ABCD_weekly_activity_scales_alldays$fit_ss_perday_veryactivemin)

ABCD_weekly_activity_scales_alldays$fit_ss_perday_fairlyactivemin<-as.numeric(ABCD_weekly_activity_scales_alldays$fit_ss_perday_fairlyactivemin)

ABCD_weekly_activity_scales_alldays$fit_ss_fitbit_rest_hr<-as.numeric(ABCD_weekly_activity_scales_alldays$fit_ss_fitbit_rest_hr)

#### Data wrangling for Aim 1 ####

# Using largest time point T2 and not covid time point, or smaller baseline sample

ABCD_weekly_activity_scales_alldays%>%filter(ABCD_weekly_activity_scales_alldays$eventname=="2_year_follow_up_y_arm_1")->ABCD_weekly_activity_scales_yr2

## make an average of 3 week average

ABCD_weekly_activity_scales_yr2_GrandMean<-ABCD_weekly_activity_scales_yr2 %>% # Specify data frame

group_by(subjectkey) %>% # Specify group indicator

summarise_at(vars(c("interview_age","fit_ss_perday_sedentarymin","fit_ss_perday_veryactivemin", "fit_ss_perday_fairlyactivemin", "fit_ss_fitbit_rest_hr")), # Specify column

list(name = mean)) # Specify function

#Make Moderate to Intense exercise variable

ABCD_weekly_activity_scales_yr2_GrandMean$totalminmoderteactivity<-ABCD_weekly_activity_scales_yr2_GrandMean$fit_ss_perday_fairlyactivemin_name+ABCD_weekly_activity_scales_yr2_GrandMean$fit_ss_perday_veryactivemin_name

### PPS/BMI total scores from DEAP

PPS_demo <- read_csv("DEAP-data-download.csv",na = "NA")

PPS_demo[,c("src_subject_id","event_name","rel_family_id","sex_at_birth","household.income","race_ethnicity","race.4level", "race.6level", "anthro_bmi_calc","prodrom_psych_ss_severity_score","prodrom_psych_ss_severity_score_nm")]->PPSEXTINT_demo

#define numeric vs factor

PPS_demo$rel_family_id<-as.factor(PPS_demo$rel_family_id)

PPS_demo$household.income<-as.factor(PPS_demo$household.income)

PPS_demo$sex_at_birth<-as.factor(PPS_demo$sex_at_birth)

PPS_demo$prodrom_psych_ss_severity_score<-as.numeric(PPS_demo$prodrom_psych_ss_severity_score)

### PPS/EXT/INT total scores from NDA

EXTINT_nda <- read_csv("abcd_cbcls01.csv")

EXTINT_nda[,c("subjectkey","eventname","cbcl_scr_dsm5_depress_nm","cbcl_scr_dsm5_depress_t","cbcl_scr_dsm5_anxdisord_nm","cbcl_scr_dsm5_anxdisord_t","cbcl_scr_syn_internal_nm", "cbcl_scr_syn_internal_t","cbcl_scr_syn_external_t", "cbcl_scr_syn_external_nm" )]->EXTINT_nda_MDDANX

# exclude individuals that are missing symptom items

EXTINT_nda_MDDANX$cbcl_scr_dsm5_anxdisord_t[EXTINT_nda_MDDANX$cbcl_scr_dsm5_anxdisord_nm=="9"]<- ""

EXTINT_nda_MDDANX$cbcl_scr_dsm5_depress_t[EXTINT_nda_MDDANX$cbcl_scr_dsm5_depress_nm=="13"]<- ""

EXTINT_nda_MDDANX$cbcl_scr_syn_internal_t[EXTINT_nda_MDDANX$cbcl_scr_syn_internal_nm=="32"]<- ""

EXTINT_nda_MDDANX$cbcl_scr_syn_external_t[EXTINT_nda_MDDANX$cbcl_scr_syn_external_nm=="35"]<- ""

EXTINT_nda_MDDANX$cbcl_scr_syn_external_t[EXTINT_nda_MDDANX$cbcl_scr_syn_external_nm=="1"]<- ""

# define variables as numeric

EXTINT_nda_MDDANX$cbcl_scr_syn_external_t<-as.numeric(EXTINT_nda_MDDANX$cbcl_scr_syn_external_t)

EXTINT_nda_MDDANX$cbcl_scr_dsm5_anxdisord_t<-as.numeric(EXTINT_nda_MDDANX$cbcl_scr_dsm5_anxdisord_t)

EXTINT_nda_MDDANX$cbcl_scr_dsm5_depress_t<-as.numeric(EXTINT_nda_MDDANX$cbcl_scr_dsm5_depress_t)

EXTINT_nda_MDDANX$cbcl_scr_syn_internal_t<-as.numeric(EXTINT_nda_MDDANX$cbcl_scr_syn_internal_t)

EXTINT_nda_MDDANX$cbcl_scr_syn_external_t<-as.numeric(EXTINT_nda_MDDANX$cbcl_scr_syn_external_t)

#### Aim 1: Activity and Health metrics map onto current symptoms? (all year 2) ####

#### Year 2 data wrangling ####

# Filter to be just year 2 and merge files

PPS_demo%>%filter(PPS_demo$event_name=="2_year_follow_up_y_arm_1")->PPS_demo_y2

EXTINT_nda_MDDANX%>%filter(EXTINT_nda_MDDANX$eventname=="2_year_follow_up_y_arm_1")->EXTINT_nda_MDDANX_y2

PPS_demo_y2$src_subject_id->PPS_demo_y2$subjectkey

Y2_fitbit_symptoms <- merge(PPS_demo_y2[,c("src_subject_id","sex_at_birth","race_ethnicity","rel_family_id","race.6level", "anthro_bmi_calc","subjectkey","prodrom_psych_ss_severity_score")],ABCD_weekly_activity_scales_yr2_GrandMean[,c("subjectkey","interview_age_name","fit_ss_fitbit_rest_hr_name","fit_ss_perday_sedentarymin_name","totalminmoderteactivity")], by ="subjectkey")

Y2_fitbit_symptoms <- merge(EXTINT_nda_MDDANX_y2,Y2_fitbit_symptoms, by ="subjectkey")

PPSEXTINT_demo%>%filter(PPSEXTINT_demo$event_name=="baseline_year_1_arm_1")->PPSEXTINT_demo_householdincome

PPSEXTINT_demo_householdincome$src_subject_id->PPSEXTINT_demo_householdincome$subjectkey

Y2_fitbit_symptoms <- merge(PPSEXTINT_demo_householdincome[,c("subjectkey","household.income")],Y2_fitbit_symptoms, by ="subjectkey")

#redefine the factors and numeric variables

Y2_fitbit_symptoms$rel_family_id<-as.factor(Y2_fitbit_symptoms$rel_family_id)

Y2_fitbit_symptoms$household.income<-as.factor(Y2_fitbit_symptoms$household.income)

Y2_fitbit_symptoms$sex_at_birth<-as.factor(Y2_fitbit_symptoms$sex_at_birth)

Y2_fitbit_symptoms$interview_age<-as.numeric(Y2_fitbit_symptoms$interview_age)

Y2_fitbit_symptoms$prodrom_psych_ss_severity_score<-as.numeric(Y2_fitbit_symptoms$prodrom_psych_ss_severity_score)

Y2_fitbit_symptoms$cbcl_scr_syn_external_t<-as.numeric(Y2_fitbit_symptoms$cbcl_scr_syn_external_t)

Y2_fitbit_symptoms$cbcl_scr_dsm5_anxdisord_t<-as.numeric(Y2_fitbit_symptoms$cbcl_scr_dsm5_anxdisord_t)

Y2_fitbit_symptoms$cbcl_scr_dsm5_depress_t<-as.numeric(Y2_fitbit_symptoms$cbcl_scr_dsm5_depress_t)

Y2_fitbit_symptoms$cbcl_scr_syn_internal_t<-as.numeric(Y2_fitbit_symptoms$cbcl_scr_syn_internal_t)

Y2_fitbit_symptoms$cbcl_scr_syn_external_t<-as.numeric(Y2_fitbit_symptoms$cbcl_scr_syn_external_t)

Y2_fitbit_symptoms$anthro_bmi_calc<-as.numeric(Y2_fitbit_symptoms$anthro_bmi_calc)

### Make log transformations of symptom scores to address high number of zero scores

#Y2_fitbit_symptoms$prodrom_psych_ss_severity_score_natlog<-log(Y2_fitbit_symptoms[,c("prodrom_psych_ss_severity_score")]+1)

#Y2_fitbit_symptoms$prodrom_psych_ss_severity_score_log10<-log10(Y2_fitbit_symptoms[,c("prodrom_psych_ss_severity_score")]+1)

#Y2_fitbit_symptoms$cbcl_scr_dsm5_anxdisord_t_natlog<-log((Y2_fitbit_symptoms[,c("cbcl_scr_dsm5_anxdisord_t")]+1))

#Y2_fitbit_symptoms$cbcl_scr_dsm5_anxdisord_t_log10<-log10((Y2_fitbit_symptoms[,c("cbcl_scr_dsm5_anxdisord_t")]+1))

#Y2_fitbit_symptoms$cbcl_scr_dsm5_depress_t_natlog<-log((Y2_fitbit_symptoms[,c("cbcl_scr_dsm5_depress_t")]+1))

#Y2_fitbit_symptoms$cbcl_scr_dsm5_depress_t_log10<-log10((Y2_fitbit_symptoms[,c("cbcl_scr_dsm5_depress_t")]+1))

#Y2_fitbit_symptoms$cbcl_scr_syn_internal_t_natlog<-log((Y2_fitbit_symptoms[,c("cbcl_scr_syn_internal_t")]+1))

#Y2_fitbit_symptoms$cbcl_scr_syn_internal_t_log10<-log10((Y2_fitbit_symptoms[,c("cbcl_scr_syn_internal_t")]+1))

#Y2_fitbit_symptoms$cbcl_scr_syn_external_t_natlog<-log((Y2_fitbit_symptoms[,c("cbcl_scr_syn_external_t")]+1))

#Y2_fitbit_symptoms$cbcl_scr_syn_external_t_log10<-log10((Y2_fitbit_symptoms[,c("cbcl_scr_syn_external_t")]+1))

#### Aim 1 Models ####

RHR_Aim1<-lmer(fit_ss_fitbit_rest_hr_name~prodrom_psych_ss_severity_score+cbcl_scr_syn_internal_t+cbcl_scr_syn_external_t+interview_age+sex_at_birth+anthro_bmi_calc+household.income+(1|rel_family_id),data=Y2_fitbit_symptoms)

summary(RHR_Aim1,control = lmerControl(optimizer ="Nelder Mead"))

vif(RHR_Aim1)

Aim1_PLEs_RHR<-ggplot(Y2_fitbit_symptoms, aes(x=prodrom_psych_ss_severity_score, y=fit_ss_fitbit_rest_hr_name)) +geom_point( color="#f94144") + xlab("PQ-CB Symptom Severity Score")+ylab("Resting Heart Rate")+ labs( title = 'A.') + geom_smooth(method=lm,color="#f94144", se=TRUE)

Aim1_INT_RHR<-ggplot(Y2_fitbit_symptoms, aes(x=cbcl_scr_syn_internal_t, y=fit_ss_fitbit_rest_hr_name)) +geom_point( color="#f9c74f") + xlab("CBCL Total Internalizing Score (t-score)")+ylab("Resting Heart Rate")+ labs( title = 'D.') + geom_smooth(method=lm,color="#f9c74f", se=TRUE)

Aim1_EXT_RHR<-ggplot(Y2_fitbit_symptoms, aes(x=cbcl_scr_syn_external_t, y=fit_ss_fitbit_rest_hr_name)) +geom_point( color="#4d908e") + xlab("CBCL Total Internalizing Score (t-score)")+ylab("Resting Heart Rate")+ labs( title = 'G.') + geom_smooth(method=lm,color="#4d908e", se=TRUE)

Sed_Aim1<-lmer(fit_ss_perday_sedentarymin_name~prodrom_psych_ss_severity_score+cbcl_scr_syn_internal_t+cbcl_scr_syn_external_t+interview_age+sex_at_birth+anthro_bmi_calc+household.income+(1|rel_family_id),data=Y2_fitbit_symptoms)

summary(Sed_Aim1, control = lmerControl(optimizer ="Nelder Mead"))

vif(Sed_Aim1)

Aim1_PLEs_Sed<-ggplot(Y2_fitbit_symptoms, aes(x=prodrom_psych_ss_severity_score, y=fit_ss_perday_sedentarymin_name)) +geom_point( color="#f3722c") + xlab("PQ-CB Symptom Severity Score")+ylab("Total Minutes Sedentary")+ labs( title = 'B.') + geom_smooth(method=lm,color="#f3722c", se=TRUE)

Aim1_INT_Sed<-ggplot(Y2_fitbit_symptoms, aes(x=cbcl_scr_syn_internal_t, y=fit_ss_perday_sedentarymin_name)) +geom_point( color="#90be6d") + xlab("CBCL Total Internalizing Score (t-score)")+ylab("Minutes of Sedentary Activity")+ ylim(0,1500)+ labs( title = 'E.') + geom_smooth(method=lm,color="#90be6d", se=TRUE)

Aim1_EXT_Sed<-ggplot(Y2_fitbit_symptoms, aes(x=cbcl_scr_syn_external_t, y=fit_ss_perday_sedentarymin_name)) +geom_point( color="#277da1") + xlab("CBCL Total Externalizing Score (t-score)")+ylab("Minutes of Sedentary Activity")+ labs( title = 'H.') + geom_smooth(method=lm,color="#277da1", se=TRUE)

Mod_Aim1<-lmer(totalminmoderteactivity~prodrom_psych_ss_severity_score+cbcl_scr_syn_internal_t+cbcl_scr_syn_external_t+interview_age+sex_at_birth+anthro_bmi_calc+household.income+(1|rel_family_id),data=Y2_fitbit_symptoms)

summary(Mod_Aim1, control = lmerControl(optimizer ="Nelder Mead"))

vif(Mod_Aim1)

Aim1_PLEs_Mod<-ggplot(Y2_fitbit_symptoms, aes(x=prodrom_psych_ss_severity_score, y=totalminmoderteactivity)) +geom_point( color="#f8961e") + xlab("PQ-CB Symptom Severity Score")+ylab("Total Minutes Physically Active")+ labs( title = 'C.') + geom_smooth(method=lm,color="#f8961e", se=TRUE)

Aim1_INT_Mod<-ggplot(Y2_fitbit_symptoms, aes(x=cbcl_scr_syn_internal_t, y=totalminmoderteactivity)) +geom_point( color="#43aa8b") + xlab("CBCL Total Internalizing Score (t-score)")+ylab("Minutes of Physically Active")+ labs( title = 'F.') + geom_smooth(method=lm,color="#43aa8b", se=TRUE)

Aim1_EXT_Mod<-ggplot(Y2_fitbit_symptoms, aes(x=cbcl_scr_syn_external_t, y=totalminmoderteactivity)) +geom_point( color="#6a4c93") + xlab("CBCL Total Externalizing Score (t-score)")+ylab("Minutes Physically Active")+ labs( title = 'I.') + geom_smooth(method=lm,color="#6a4c93", se=TRUE)

figure1<-grid.arrange(Aim1_PLEs_RHR, Aim1_PLEs_Sed, Aim1_PLEs_Mod, Aim1_INT_RHR, Aim1_INT_Sed, Aim1_INT_Mod, Aim1_EXT_RHR, Aim1_EXT_Sed, Aim1_EXT_Mod, nrow = 3, ncol=3)

#### Follow Up Analyses ####

as.numeric(Y2_fitbit_symptoms$cbcl_scr_dsm5_depress_t)-> Y2_fitbit_symptoms$cbcl_scr_dsm5_depress_t

as.numeric(Y2_fitbit_symptoms$cbcl_scr_dsm5_anxdisord_t)-> Y2_fitbit_symptoms$cbcl_scr_dsm5_anxdisord_t

RHR_FU1<-lmer(fit_ss_fitbit_rest_hr_name~prodrom_psych_ss_severity_score+cbcl_scr_dsm5_depress_t+cbcl_scr_dsm5_anxdisord_t++cbcl_scr_syn_external_t+interview_age+sex_at_birth+anthro_bmi_calc+household.income+(1|rel_family_id),data=Y2_fitbit_symptoms)

summary(RHR_FU1)

vif(RHR_FU1)

Mod_FU<-lmer(totalminmoderteactivity~prodrom_psych_ss_severity_score+cbcl_scr_dsm5_depress_t+cbcl_scr_dsm5_anxdisord_t+cbcl_scr_syn_external_t+interview_age+sex_at_birth+anthro_bmi_calc+household.income+(1|rel_family_id),data=Y2_fitbit_symptoms)

summary(Mod_FU)

vif(Mod_FU)

#### demographics ####

table(Y2_fitbit_symptoms$sex_at_birth)

table(Y2_fitbit_symptoms$race.6level)

table(Y2_fitbit_symptoms$household.income)

mean(Y2_fitbit_symptoms$interview_age)

sd(Y2_fitbit_symptoms$interview_age)

mean(Y2_fitbit_symptoms$anthro_bmi_calc)

sd(Y2_fitbit_symptoms$anthro_bmi_calc)

####Effect Size Graphs ####

library(metaviz)

library(readxl)

library(effectsize)

#calculated the d and CI using t_to_d with effectsize

#

Fitness_Metric<-c("Resting Heart Rate (RHR)","Resting Heart Rate (RHR)","Resting Heart Rate (RHR)", "Sedentary", "Sedentary", "Sedentary", "Vigorous Physical Activity", "Vigorous Physical Activity","Vigorous Physical Activity")

Symptom_Dimension<-c("PQ-CB Symptom Severity", "CBCL Internalizing Total", "CBCL Externalizing Total","PQ-CB Symptom Severity", "CBCL Internalizing Total", "CBCL Externalizing Total","PQ-CB Symptom Severity", "CBCL Internalizing Total", "CBCL Externalizing Total")

d<-c("0.06000","0.11000","0.05000","0.16000","-0.00541","-0.00039","-0.08000","-0.19000","0.06000")

ci95<-c("0.06", "0.06","0.06","0.06","0.05541","0.06039","0.06","0.06","0.06")

se<-c("0.02", "0.02","0.02","0.02","0.02","0.02","0.02","0.02","0.02")

transdiag_effect<-data.frame(Fitness_Metric,Symptom_Dimension,d,ci95,se)

as.numeric(transdiag_effect$d)->transdiag_effect$d

as.numeric(transdiag_effect$ci95)->transdiag_effect$ci95

as.factor(transdiag_effect$Fitness_Metric)->transdiag_effect$Fitness_Metric

as.factor(transdiag_effect$Symptom_Dimension)->transdiag_effect$Symptom_Dimension

as.numeric(transdiag_effect$se)->transdiag_effect$se

Figure2<-viz_forest(x = transdiag_effect[1:9, c("d", "se")],

group = transdiag_effect[1:9, "Fitness_Metric"],

study_labels = transdiag_effect[1:9, "Symptom_Dimension"],

summary_label = c("Summary Resting Heart Rate", "Summary Sedentary", "Summary Vigorous Physical Activity"),

xlab = "Cohen's d",

col = c("#d00000", "#e85d04", "#faa307")[transdiag_effect[1:9, "Fitness_Metric"]],

summary_col = c("#d00000", "#e85d04", "#faa307"))

Fitness_Metric2<-c("Resting Heart Rate (RHR)","Resting Heart Rate (RHR)","Resting Heart Rate (RHR)", "Resting Heart Rate (RHR)","Physical Activity", "Physical Activity","Physical Activity","Physical Activity")

Symptom_Dimension2<-c("PQ-CB Symptom Severity", "CBCL Depression Total", "CBCL Anxiety Total","CBCL Externalizing Total","PQ-CB Symptom Severity", "CBCL Depression Total", "CBCL Anxiety Total","CBCL Externalizing Total")

d2<-c("0.06000","0.09","0.04000","0.04","-0.08","-0.12","-0.07000","0.07")

ci952<-c("0.04", "0.04","0.04","0.04","0.04","0.04","0.04","0.04")

se2<-c("0.02", "0.02","0.02","0.02","0.02","0.02","0.02","0.02")

transdiag_effect2<-data.frame(Fitness_Metric2,Symptom_Dimension2,d2,ci952,se2)

as.numeric(transdiag_effect2$d2)->transdiag_effect2$d2

as.numeric(transdiag_effect2$ci952)->transdiag_effect2$ci952

as.factor(transdiag_effect2$Fitness_Metric2)->transdiag_effect2$Fitness_Metric2

as.factor(transdiag_effect2$Symptom_Dimension2)->transdiag_effect2$Symptom_Dimension2

as.numeric(transdiag_effect2$se2)->transdiag_effect2$se2

Figure3<-viz_forest(x = transdiag_effect2[1:8, c("d2", "se2")],

group = transdiag_effect2[1:8, "Fitness_Metric2"],

study_labels = transdiag_effect2[1:8, "Symptom_Dimension2"],

summary_label = c("Summary Resting Heart Rate", "Summary Sedentary", "Summary Vigorous Physical Activity"),

xlab = "Cohen's d",

col = c("#38b000", "#4895ef")[transdiag_effect2[1:8, "Fitness_Metric2"]],

summary_col = c("#38b000", "#4895ef"))
